# A Novel Methylation Marker NRN1 plus TERT and FGFR3 Mutation Using Urine Sediment Enables the Detection of Urothelial Bladder Carcinoma

**DOI:** 10.3390/cancers15030615

**Published:** 2023-01-19

**Authors:** Junjie Zhang, Ran Xu, Qiang Lu, Zhenzhou Xu, Jianye Liu, Pei Li, Yaqun Zhang, Chuanchi Zhou, Lufeng Luo, Wei Tang, Zhenting Wang, Manman Cao, Jian Cao, Genming Xu, Long Wang

**Affiliations:** 1 Department of Urology, The Third Xiangya Hospital, Central South University, Changsha 410013, China; 2 Department of Thoracic Surgery, Xiangya Hospital, Central South University, Changsha 410008, China; 3 Department of Urology, The Second Xiangya Hospital, Central South University, Changsha 410028, China; 4 Department of Urology, Hunan Provincial People’s Hospital, First Affiliated Hospital of Hunan Normal University, Changsha 410002, China; 5 Department of Urology, Hunan Cancer Hospital, The Affiliated Cancer Hospital of Xiangya School of Medical, Central South University, Changsha 410013, China; 6 Hunan Yearth Biotechnology Co., Ltd., Changsha 410205, China; 7 Department of Urology, Beijing Hospital, National Center of Gerontology, National Health Commission, Institute of Geriatric Medicine, Chinese Academy of Medical Sciences, Beijing 100006, China; 8 Affiliated Haikou Hospital of Xiangya Medical College, Central South University, Changsha 410017, China

**Keywords:** urothelial bladder carcinoma, diagnostic tool, SNPs, DNA methylation region

## Abstract

**Simple Summary:**

In the present study, we aimed to construct a methylation diagnostic tool using urine sediment for the detection of urothelial bladder carcinoma (UBC), and improved the diagnostic performance of the model by incorporating single nucleotide polymorphism sites. In stage I, single NRN1 exhibited the highest AUC. At the best cutoff value of 5.16, single NRN1 biomarker showed a sensitivity of 0.93 and specificity of 0.97. In stage II, random forest algorithm was applied to construct the model, including NRN1, TERT C228T and FGFR3 p.S249C. The tool exhibited AUC values of 0.953, 0.946 and 0.951 in training, test and all cohort. In the external validation cohort (stage III), the model achieved an AUC of 0.935, sensitivity of 0.864 and specificity of 0.895. The model also exhibited a superior sensitivity and comparable specificity compared with conventional cytology and FISH, and may be used as a replaceable approach for the detection of UBC.

**Abstract:**

Background: Aberrant DNA methylation is an early event during tumorigenesis. In the present study, we aimed to construct a methylation diagnostic tool using urine sediment for the detection of urothelial bladder carcinoma, and improved the diagnostic performance of the model by incorporating single-nucleotide polymorphism (SNP) sites. Methods: A three-stage analysis was carried out to construct the model and evaluate the diagnostic performance. In stage I, two small cohorts from Xiangya hospital were recruited to validate and identify the detailed regions of collected methylation biomarkers. In stage II, proof-of-concept study cohorts from the Hunan multicenter were recruited to construct a diagnostic tool. In stage III, a blinded cohort comprising suspicious UBC patients was recruited from Beijing single center to further test the robustness of the model. Results: In stage I, single NRN1 exhibited the highest AUC compared with six other biomarkers and the Random Forest model. At the best cutoff value of 5.16, a single NRN1 biomarker gave a diagnosis with a sensitivity of 0.93 and a specificity of 0.97. In stage II, the Random Forest algorithm was applied to construct a diagnostic tool, consisting of NRN1, TERT C228T and FGFR3 p.S249C. The tool exhibited AUC values of 0.953, 0.946 and 0.951 in training, test and all cohorts. At the best cutoff value, the model resulted in a sensitivity of 0.871 and a specificity of 0.947. In stage III, the diagnostic tool achieved a good discrimination in the external validation cohort, with an overall AUC of 0.935, sensitivity of 0.864 and specificity of 0.895. Additionally, the model exhibited a superior sensitivity and comparable specificity compared with conventional cytology and FISH. Conclusions: The diagnostic tool exhibited a highly specific and robust performance. It may be used as a replaceable approach for the detection of UBC.

## 1. Background

Urothelial bladder carcinoma (UBC) is one of the most common malignancies of the urinary tract, with approximately an estimated 573,278 new cases and 212,536 deaths per year worldwide [1]. Typical diagnosis and surveillance of UBC involve the use of cystoscopy, cytology and FISH [2,3]. Cystoscopy is regarded as the gold standard for the detection of UBC, which exhibits relatively high clinical sensitivity but low patient acceptance owing to its invasive nature [4]. In contrast, urine cytology and FISH are noninvasive and specific, but lack sensitivity, especially in low-grade tumors. These facts, together with the high cost and follow-up biopsy procedures, have led to many attempts to develop alternative noninvasive methods to detect UBC.

DNA methylation is one of the epigenetic mechanisms regulating gene expression [5,6]. Increased methylation of tumor-associated suppressor genes is an early event in many tumors. This may indicate that altered DNA methylation patterns could be one of the first detectable neoplastic changes associated with tumorigenesis and assist the detection of cancer [7]. Currently, several commercial methylation-based biomarkers have been successfully incorporated into available in vitro diagnostic (IVD) devices. For colorectal cancer screening, a methylation marker panel consisting of bone morphogenic protein 3 gene (BMP3) and NDRG family member 4 gene (NDRG4) has been developed and validated in multi-prospective screening trials [8]. For UBC, a kit named Bladder EpiCheck^TM^ used for recurrence monitoring was made possible with the assessment of 15 methylation biomarkers [9].

In the present study, we screened seven methylation biomarkers from previous studies or databases, including PSMD14, AKAP13, ZNF184, cg16966315, NRN1, P14ARF and MEIS1 [10,11,12]. We carried out a three-stage analysis to construct and evaluate the diagnostic performance of the panel, using urine sediment. Firstly, we identified the top six regions of each methylation biomarkers (cohort 1; *n* = 52) using NGS and verified the biomarkers (cohort 2; *n* = 69) using MS-PCR. Secondly, we constructed a diagnostic panel using the proof-of-concept study cohort (cohort 3; *n* = 240). Additionally, we tried to improve the diagnostic performance of the panel by incorporating two mutation biomarkers. Finally, we performed external validation of the diagnostic model using a blinded cohort (cohort 4; *n* = 82), comprising suspicious UBC patients showing symptoms.

## 2. Materials and Methods

### 2.1. Study Cohort and Ethics Statement

This study was registered in http://www.chictr.org.cn/, accessed on 18 February 2020, with the number of ChiCTR2000029980. All participants were recruited as approved by the Ethics Committee of Xiangya Hospital (Changsha, Hunan), The Second Xiangya Hospital (Changsha, Hunan), Hunan Provincial People’s Hospital (Changsha, Hunan), Hunan Cancer Hospital (Changsha, Hunan) and Beijing Hospital (Beijing, Beijing) after written informed consents were obtained. Studies were conducted in accordance with the ethical principles in the Declaration of Helsinki.

In total, 443 hematuria patients were prospectively recruited from five centers, including 271 UBC patients and 172 patients with benign urological diseases. Comprehensive examination, including ultrasound and CT, was performed in all patients. Pathological diagnosis was set as the gold standard.

### 2.2. Study Design

In stage I, we identified seven UBC-specific methylation biomarkers by comprehensive analysis cancer cell line(CCLE) database and reported studies [10,11,12]. Next-generation sequencing (NGS) was applied in cohort 1 to validate the reported biomarkers and identify the detailed methylation region. As our aim was to develop a tool that could be easily incorporated into routine clinical practice, the methylation region of different biomarkers, with the most significant adjusted *p* values and the highest area under the curve (AUC) values, were selected for MS-PCR validation and further screening in cohort 2. Additionally, the diagnostic model was preliminarily established in this cohort using MS-PCR data.

In stage II, we recruited a total of 240 participants from Hunan multicenter for further validation the model. MS-PCR was applied to detect methylation sites screened in cohort 2, and cycle threshold (Ct) values were obtained. For further optimization of the model, several single nucleotide polymorphisms (SNPS) from 5 genes associated with UBC were detected in cohort 3 using the NGS technique. Quantitative polymerase chain reaction (qPCR) was further used for the point mutation detection. All patients in cohort 3 were randomly divided into training and test cohorts. The Random Forest algorithm was applied to reconstruct an improved diagnostic model.

In stage III, 82 suspicious UBC participants were recruited from the Beijing single center, and the stability and reproducibility of the panel were evaluated and compared with cytology and fluorescence in situ hybridization (FISH).

### 2.3. Sample Collection, DNA Isolation and Sodium Bisulfite Conversion

For all participants, each urine sample (at least 30 mL) was collected from the first miction in the morning. The urine samples were centrifuged at 1600× *g* for 10 min at 4 °C, the supernatant was discarded and the pellet was carefully collected into new vacant 2 mL tubes. The same procedure was performed again at 12,000× *g* for 10 min at 25 °C. Then, 200 μL of 1× PBS was added to each tube to resuspend the cells. DNA isolation was performed using the Tissue Genomic DNA Extraction Kit (cat DP304, Tiangen Biotechnology, Beijing, China) according to the manufacture’s instruction. In total, 200~300 ng of genomic DNA from each sample was treated with sodium bisulfite with the EZ DNA Methylation-Lightning Kits (Cat D5031, Zymo Research, USA).

### 2.4. Library Preparation and Sequencing

The PCR of selected methylation sites or SNP was conducted using 3 ul of converted or unconverted DNA, 400 mM specific forward and reverse primers, and 25 μL 2× Glod 360 Master MIX (Applied Biosystems, Cat: 4398886) in a total volume of 50 uL. After one round purification with 1.2× AMPure XP beads (Beckman), the ligased-product underwent one more PCR-amplification using 400 nM P5/INDEX primers and 25 μL HIFI Master MIX (Kapa biosystems, Cat: KK2602). After another round of purification using 1× AMPure beads, the final library pool was quantified by ABI 7500 fast Real-Time PCR system (Applied Biosystems) and sequenced on a NextSeq 500 system (Illumina, USA) to obtain paired-end 150 bp reads.

### 2.5. MS-PCR

Sodium bisulfite conversion and purification of 100 ng genomic DNA were performed using EZ DNA Methylation Lightning TM Kit (Zymo Research Corporation, Irvine, CA, USA), according to the manufacturer’s protocol. ACTB was set as the internal reference. Ct values represented the relative methylation quantity of CpG markers and the internal reference gene (ACTB), which was measured by FAM and VIC signals separately.

### 2.6. RT-qPCR

Briefly, a PCR procedure was used to amplify the proximal TERT promoter nucleotide positions chr5:1295228 C > T and FGFR3 chr4: 1803568 C > G referred to as TERT C228T and FGFR3 p.S249C. GAPDH was set as the internal reference. Ct values represented the relative quantity of the detected biomarker and the internal reference gene (GAPDH), which was measured by FAM and VIC signals separately.

### 2.7. Statistical Analysis

The model performance was evaluated by the area under the curve (AUC) statistics. The classifier of the AUC analysis was methylation ratio of biomarkers or Δct value of biomarkers in each sample. Chi-square test was used for categorical variables; a t-test was used for continuous variables and the Mann–Whitney U test for non-normally distributed variable data. The primary end points were AUC values, sensitivity and specificity of the model. All hypothesis tests were two-sided with a *p* value < 0.05 considered to be statistically significant. All statistical analysis and data visualizations were carried out in R software (R version 3.4.3) and GraphPad Prism 8 (version 8.0.2). Adobe Illustrator (CC 2017) was used for image processing.

## 3. Results

### 3.1. Baseline Characteristics and Flow Chart

**Figure 1:**
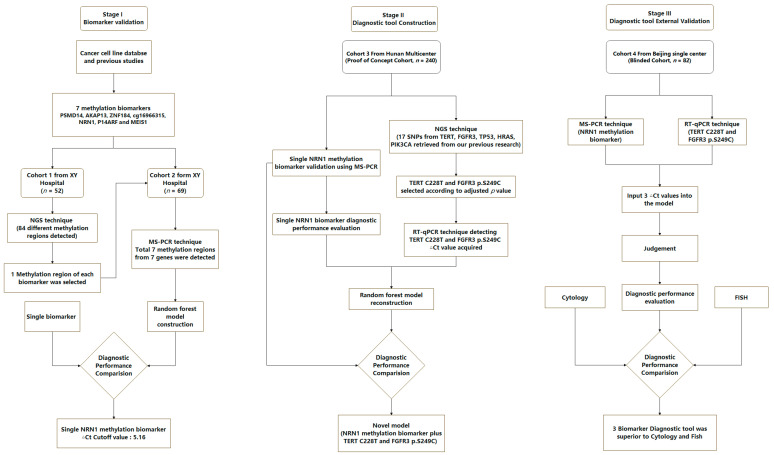
Flow chart of the study design.

The baseline characteristics of cohort 1~2 were shown in Table S1. The baseline characteristics of cohort 3~4 were shown in Table 1. The exact flow chart of the present study is summarized in Figure 1.

### 3.2. Validation of the Seven Methylation Biomarkers Using NGS and MS-PCR

**Figure 2:**
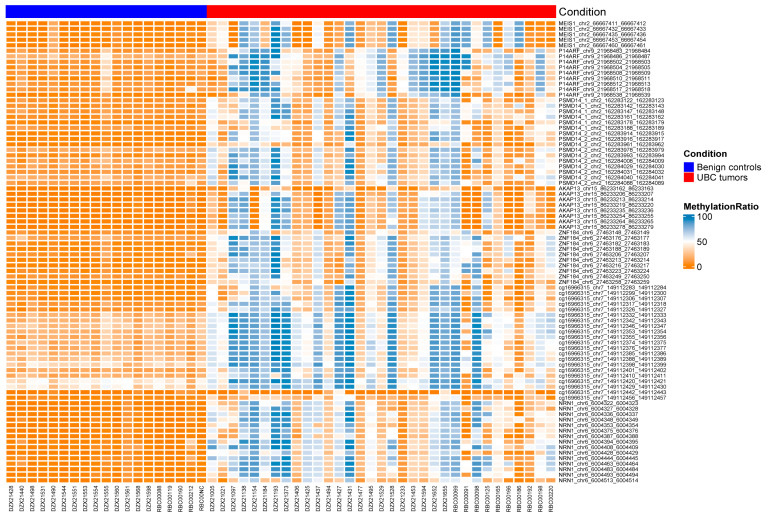
The heatmap of different methylation regions in cohort 1. The parameter “condition” represents the benign controls in blue and the UBC tumor samples in red.

Cohort 1 was used to carry out a preliminary validation study, in which different methylation regions were detected. This revealed 84 methylation regions of 7 biomarkers with statistically significant differential expression between tumor (*n* = 33) and normal (*n* = 19) from Xiangya hospital (Figure 2). According to the AUC values, we selected top 6 regions of each methylation biomarker to list in Table 2 (MEIS1 with total 5 regions). Primers was designed for the regions with the most significant adjusted *p* values of each biomarker for MS-PCR validation (Table S2).

**Figure 3:**
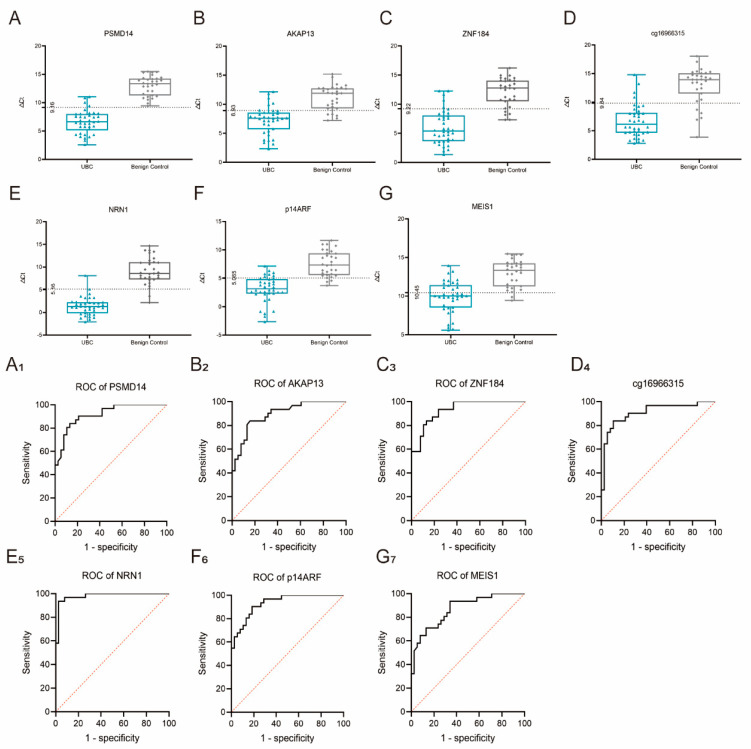
The box plot and AUC of different methylation biomarkers in cohort 2 by detecting methylation regions. (**A**–**G**) represents the ΔCt value distributions of each biomarker in UBC and control groups. (**A1**–**G7**) represents the AUC values of each biomarker. The classifier of the AUC analysis was Δct value of biomarkers in each sample.

We next recruited an independent Xiangya cohort of 38 UBC patients and 31 benign controls to verify the 7 regions of these biomarkers using the MS-PCR technique in cohort 2, and found that all were differentially methylated (Figure 3). NRN1 exhibited the highest AUC among the 7 biomarkers, whereas MEIS1 demonstrated the lowest AUC, with an overall value of 0.9796 and 0.8680, respectively. At the best cutoff value of 5.16, the single NRN1 biomarker gave a diagnosis with a sensitivity of 0.93 and a specificity of 0.97.

We then used the Boruta feature selection algorithm to rank the 7 methylation biomarkers by their importance and Random Forest algorithm to highlight the most powerful combinations for distinguishing UBC from benign controls in cohort 2. NRN1 and P14ARF were selected for construction of the panel. However, the model only achieved an AUC of 0.9397 (Figure S1), which was not better than the single NRN1 biomarker. Thus, this model was abandoned, and the single NRN1 biomarker was chosen for Hunan multicenter validation.

To further improve the data evidence, we also detected the mRNA expression of NRN1 in tissue levels. Firstly, we used 32 previously collected frozen tumor tissues and 10 control tissues to detect the relative expression of NRN1 using RT-qPCR. The tumor tissues exhibited higher ΔCt values compared with the control group, with *p* < 0.01 (Figure S2A). Secondly, we collected 10 pairs of frozen tumor tissues and adjacent tissues. Similarly, tumor tissues exhibited higher ΔCt values compared with paired adjacent tissues, with *p* < 0.05 (Figure S2B). Thirdly, GEPIA2 (GEPIA 2 (cancer-pku.cn)) online database was applied to compare the NRN1 expression of TCGA and GTEx data (Figure S2C). The expression of NRN1 in the tumor group was significantly lower than that of normal group, with *p* < 0.05. Based on the above results, the expression of NRN1 mRNA level was downregulated in UBC.

### 3.3. NRN1 Methylation Biomarker plus TERT C228T and FGFR3 p.S249C. as a Diagnostic Tool to Differentiate UBC from Benign Controls

For further validation of the single NRN1 biomarker as a diagnostic tool, we recruited the proof-of-concept cohort from Hunan multicenter, including tumor (*n* = 156) and normal (*n* = 84). In cohort 3, NRN1 biomarker demonstrated an overall AUC value of 0.9391, resulting in a sensitivity of 0.85 and a specificity of 0.86 at the predefined cutoff value of 5.16 (Figure S3A,B). The diagnostic performance of NRN1 in cohort 3 decreased compared with that in cohort 2. This may be caused by the expanded study cohort.

**Figure 4:**
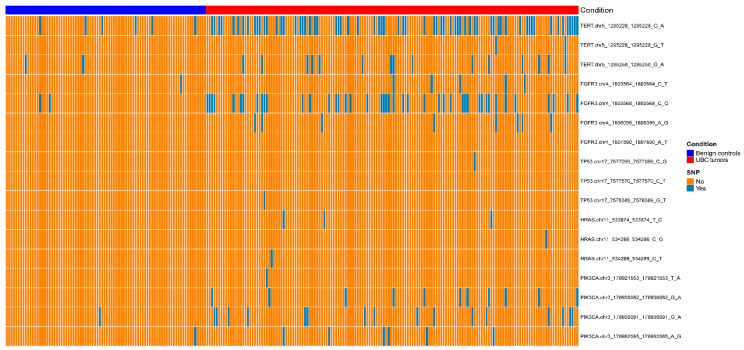
The heatmap of different SNP sites in cohort 3. The parameter “condition” represents the benign controls in blue and the UBC tumor samples in red.

For further optimization of the diagnostic tool, we retrieved five genes associated with UBC from our previous research, including TERT, FGFR3, TP53, HRAS, and PIK3CA [13]. Seventeen single nucleotide polymorphisms (SNPS) of these genes were detected in Hunan multicenter cohort using NGS technique. According to the heatmap and adjusted *p* value (Figure 4 and Table S3), only TERT C228T (228_G_A) and FGFR3 p.S249C (568_C_G) exhibited good performance in differentiating UBC from controls. Primers was designed for these two SNPs (Table S4). We then detected theΔct values of two SNPs in Hunan multicenter cohort. As shown in Figure S4 and Table S5, qPCR result of SNPs was consistent with NGS. Positive SNP detected by NGS could also be well reflected by the Δct value.

**Figure 5:**
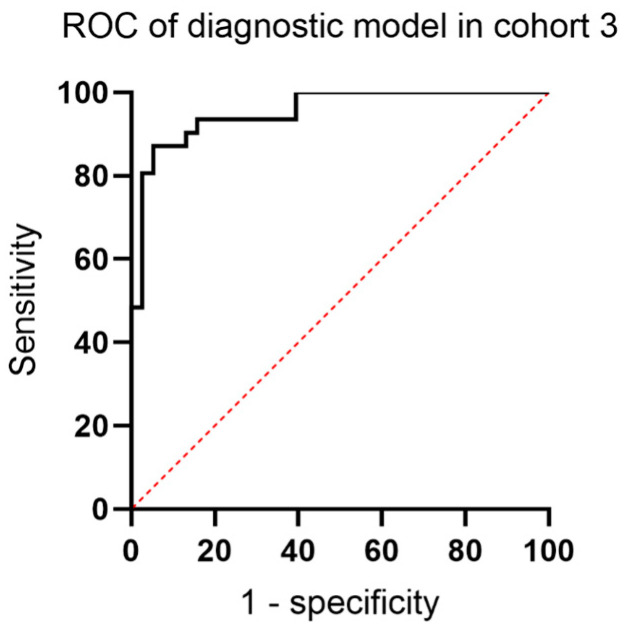
The ROC curve of diagnostic tool consisting of 1 methylation biomarker and 2 SNPs in cohort 3. The classifier of the AUC analysis was Δct value of biomarkers in each sample.

Subsequently, Δct values of NRN1, TERT C228T and FGFR3 p.S249C in Hunan multicenter cohort were applied to construct a novel panel using the Random Forest algorithm. All patients in cohort 3 were randomly divided into training and test sets with a ratio of 7:3. The novel panel exhibited high AUC values of 0.953, 0.946 and 0.951 in training, test and all cohorts (Figure 5). At the best cutoff value, the model resulted in a sensitivity of 0.871 and a specificity of 0.947.

We then assessed the model for differentiating different stages and grades of UBC. The model achieved a sensitivity of 0.916, 0.875 0.85, 0.75 and 0.80 for the detection of pTa, pT1, pT2, pT3 and pT4 tumors, respectively. Additionally, the diagnostic accuracy of the model was not affected by tumor grade, exhibiting a similar performance in detecting low- and high-grade tumors, resulting in a sensitivity of 0.865 and 0.865, respectively.

### 3.4. External Validation of the Model and Comparison with FISH and Cytology

To further test the robustness of the novel panel, an additional set of 82 suspicious UBC participants in cohort 4 was obtained from Beijing hospital to carry out external validation. The optimal model achieved a good discrimination, with an overall AUC of 0.935, sensitivity of 0.864 and specificity of 0.895 in the external cohort. With our predefined cutoff value in cohort 3, we missed only 6 out of 44 UBC cases and misdiagnosed 4 cases as BC for patients who did not have a tumor.

We investigated the diagnostic performance of the model for the detection of different stages and grades of UBC in external cohort. The model achieved a sensitivity of 0.875, 0.857 0.875, 0.75 and 1.00 for the detection of pTa, pT1, pT2, pT3 and pT4 tumors, respectively. As for the detection of low- and high-grade tumors, the model resulted in a sensitivity of 0.867 and 0.862, respectively.

We also compared the diagnostic performances of the model, cytology and FISH in cohort 4. As shown in Table 3, the overall sensitivity values of three techniques were 0.864, 0.364 and 0.682, respectively. Moreover, the overall specificity values of these techniques were 0.895, 0.895 and 0.921. This may indicate that the model exhibited a superior sensitivity and comparable specificity compared with conventional cytology and FISH.

## 4. Discussion

In recent years, several biomarkers for UBC detection have been proposed and investigated, including nuclear matrix protein (NMP22) levels and bladder tumor antigen (BTA) test. One previous study by Odea et al. directly compared NMP22, BTA and cytology for the detection of UBC using the same urine specimens [14]. Overall sensitivities of NMP22, BTA and cytology were 64%, 36% and 36%, respectively. Overall specificities of these three methods were 78%, 92% and 90%, respectively. The specificities for NMP22 were significantly lower than those of BTA and cytology, but satisfactory as a screening test. Another study from Nero et al. directly compared these three tests for the detection of stage pTa-pT1 UBC, demonstrating an overall sensitivity of 83.3%, 26.6% and 20% in pTa cases and 97.7%, 66.6% and 64.4% in pT1 cases, respectively [15]. In our external validation, the overall sensitivity of model and cytology were 0.864 and 0.364, respectively. Moreover, the overall specificity of model and cytology were 0.895 and 0.895, respectively. The diagnostic performance of cytology was consistent with previous studies. Further explorations and comparisons of the diagnostic model, NMP22 and BTA for the detection of UBC may be needed.

In the present study, single NRN1 methylation marker played the major role in the detection of UBC patients, whereas other methylation biomarkers could not enhance the diagnostic potential on this basis. In contrast, two SNPs improved the diagnostic performance when incorporated into the model. From this point of view, epigenetic and genetic biomarkers can complement and reinforce each other, resulting in a more stable and superior diagnostic performance. Kandimalla et al. demonstrated that the 3-plex methylation assay plus FGFR3 mutation exhibited a higher sensitivity and specificity compared with the methylation marker alone [16]. Another study by van Kessel et al. also confirmed that the methylation of TWIST1, ONECUT2 and OTX1 combined with mutations in FGFR3, TERT and HRAS resulted in the best overall performing panel compared with the methylation assay alone [17,18].

At present, most diagnostic techniques or devices, including cytology, FISH and cystoscopy, have difficulties in detecting early-stage UBC lesions, especially Ta tumors. Novel urine biomarkers may pave the way to solve these challenges. Wu et al., constructed a four-gene DNA methylation biomarker model, including HOXA9, PCDH17, POU4F2, and ONECUT2. The prediction model yielded an overall AUC of 0.871, with a sensitivity of 0.905 and a specificity of 0.732. As for Ta tumors, taking up nearly 71% of the entire UBC cohort, the model demonstrated a sensitivity of 0.855. Another study by van Kessel et al. constructed a six-gene methylation-mutation urine assay. The diagnostic model plus age could predict the presence of UBC with a sensitivity of 0.932, a specificity of 0.856 and an AUC of 0.96 in their cohort. As for Ta and low-grade tumors, accounting for 54% and 51% of all cases, the model showed an overall AUC of 0.93 and 0.93, respectively. In the present study, Ta and low-grade tumors took up 53.2% and 34.0% in cohort 3, respectively. The diagnostic model yielded a sensitivity of 0.916 and 0.865. Our model showed a better sensitivity for the detection of Ta and low-grade tumors compared with other two studies. This might be caused by the relatively small proportion of Ta and low-grade tumors. Moreover, the present tool consisted of only 1 methylation marker region and 2 SNPs, exhibiting significantly superior detection efficiency [19].

In the present study, the model exhibited different diagnostic performances for the detection of pathological stage Ta, T1, T2, T3 and T4 tumors, achieving a sensitivity of 0.916, 0.875 0.85, 0.75 and 0.80 in cohort 3, respectively, and 0.875, 0.857 0.875, 0.75 and 1.00 (only 1 patient) in cohort 4, respectively. The relatively low sensitivity of the model for late-stage tumors in the two cohorts may be biased and compromised by the limit number of patients. T3 and T4 patients accounted for only 8.35% of cohort 3 and 11.4% of cohort 4. In our following research, we may recruit more late-stage patients to test the diagnostic performance of the model. Another possibility was that the key gene NRN1 might be only involved in the initiation of the tumor, but not progression. The low methylation of NRN1 in late-stage tumors resulted in the decreased sensitivity of the model. However, this hypothesis needs fundamental experiments to validate. As for low- and high-grade tumors, the model showed a surprisingly consistent and stable performance in cohort 3 and 4, resulting in a sensitivity of 0.87. Based on the above-mentioned results, the model might be applied for the early detection of UBC in the future.

Previously, we investigated that urine biomarkers were effective and useful tools for the detection of UBC [13,20]. Cumulative mutation frequency of TERT promoter and FGFR3 were the most significant among the detected genes. In the present study, we further examined detailed 17 SNPs from TERT, FGFR3, TP53, HRAS, and PIK3CA in Hunan multicenter cohort. Single-SNP diagnostic model demonstrated that TERT C228T and FGFR3 p.S249C exhibited superiority compared to the other SNPs. Thus, these two SNPS were selected to incorporate into the single NRN1 model to assess the diagnostic value. Compared with the previous study, the focus of the present study shifted from genes to SNPs and methylation regions, as this was simpler and more convenient. Additionally, limited sites could be easily detected using simple techniques. This may reduce the costs and help the assay apply into clinical practice.

Neuritin 1(NRN1) was a GPI-anchored protein mainly involved in neuronal plasticity [21]. Neuritin 1 is associated with mental illness, such as schizophrenia, bipolar disorder and depression [22,23]. Recently, aberrant methylation of the *NRN1* gene promoter region has been associated with tumor development, such as gastric cancer and melanoma [24,25]. In our study, the promoter region of *NRN1* gene was first discovered as a useful biomarker to detect UBC in urine sediment. However, the biological function and methylated mechanism of NRN1 remain largely unknown, and further clarification is needed.

Hotspot mutations of TERT gene promoter regions are frequently identified in tumors or urine from UBC patients [26,27]. This mainly affected two positions, g.1295228 C > T (C228T) and g.1295250 C > T (C250T). This kind of change may alter the binding site and lead to TERT overexpression, thus further maintaining telomere length and avoiding senescence [28]. Moreover, upregulation of telomerase caused by TERT promoter mutation also contributed to tumorigenesis by promoting genomic instability [29]. FGFR3 is another frequently mutated genes detected in UBC [30]. Activating oncogenic mutations of FGFR3, including S249C, A248C, G372C, and Y375C, are predominantly identified in genetically stable tumors [31]. These mutations play an important role in cell proliferation and differentiation by activating the receptor tyrosine phosphorylation with the absence of a ligand [32].

Several limitations needed to be addressed in the present study. Firstly, the main study was performed in a case–control population. A large natural hematuria cohort is needed to validate our findings. Secondly, a number of patients were not included in the analysis process given the low DNA yields. This might be a potential factor hampering the clinical application of the diagnostic tool.

## 5. Conclusions

In summary, we developed a diagnostic tool consisting of 1 NRN1 methylation region and 2 SNPs, including TERT C228T and FGFR3 p.S249C. The model exhibited a highly specific and robust performance, and might be used as a replaceable approach for the detection of UBC.

## Figures and Tables

**Table 1:** Clinical and pathological data of the cohort.

Variables	Cohort 3 (Hunan Multicenter Cohort)		Cohort 4 (Beijing Cohort)	
	UBC	Control	UBC	Control
Sample Size	156	84	44	38
Age, Years, Median (IQR)	63 (54–70)	58 (43–69)	64(56.5–70)	57 (47–62)
Gender				
Male	114	64	34	35
Female	42	20	10	3
Smoking History				
Smoker	120	48	25	4
Non-smokers	36	36	19	34
Stage				
pTa	83	/	24	/
pT1	40		7	
pT2	20	/	18	/
pT3	8		4	
pT4	5		1	
Grade				
PUNLMP	2	/	0	/
Low grade	53	/	15	/
High grade	101	/	29	/
Tumor Type				
Primary	147	/	44	/
Recurrent	9	/	0	
Control Group	/	84	/	38
Urolithiasis	/	57	/	28
Infection	/	21	/	10
BPH	/	4	/	0
Renal cyst	/	2	/	0
TERT C228T				
Yes	66	6	20	1
No	90	78	24	37
FGFR3 p.S249C				
Yes	44	2	7	0
No	112	82	37	38
NRN1 methylation				
>5.16	133	12	37	5
<5.16	23	72	7	33

PUNLMP: papillary urothelial neoplasm of low malignant potential; UBC: urothelial bladder carcinoma; BPH: benign prostate hyperplasia.

**Table 2:** AUC values of the top 6 methylation regions of different biomarkers.

Cpg Site	AUC	Adjusted *p* Value
PSMD14_1_chr2_162283188_162283189	0.903508771929825	0.00000201
PSMD14_2_chr2_162283978_162283979	0.87719298245614	0.0000024
**PSMD14** **_2_chr2_162284029_162284030**	**0.91786283891547**	**0.000000838**
PSMD14_2_chr2_162284031_162284032	0.896331738437002	0.0000079
PSMD14_2_chr2_162284040_162284041	0.879585326953748	0.00000264
PSMD14_2_chr2_162284088_162284089	0.883078152324325	0.00000201
AKAP13_chr15_86233162_86233163	0.894736842105263	0.000319
AKAP13_chr15_86233206_86233207	0.891547049441786	0.0000163
AKAP13_chr15_86233213_86233214	0.90829346092504	0.0000165
**AKAP13_chr15_86233219_86233220**	**0.909888357256778**	**0.0000135**
AKAP13_chr15_86233264_86233265	0.893939393939394	0.0000288
AKAP13_chr15_86233278_86233279	0.890255183675756	0.0000198
ZNF184_chr6_27463148_27463149	0.968102073365231	2.91 × 10^−8^
**ZNF184_chr6_27463176_27463177**	**0.9792663476874**	**6.48 × 10^−11^**
ZNF184_chr6_27463206_27463207	0.984848484848485	1.76 × 10^−9^
ZNF184_chr6_27463216_27463217	0.986443381180223	2.91 × 10^−8^
ZNF184_chr6_27463223_27463224	0.985645933014354	1.68 × 10^−9^
ZNF184_chr6_27463258_27463259	0.976076555435456	0.000000115
cg16966315_chr7_149112306_149112307	0.910685805422648	0.000000831
**cg16966315** **_chr7_149112317_149112318**	**0.937799043062201**	**7.71 × 10^−9^**
cg16966315_chr7_149112376_149112377	0.923444976076555	1.89 × 10^−8^
cg16966315_chr7_149112410_149112411	0.90829346092504	0.000000471
cg16966315_chr7_149112420_149112421	0.87799043062201	0.00000552
cg16966315_chr7_149112429_149112430	0.84051036765397	0.0000242
NRN1_chr6_6004322_6004323	0.992025518341308	8.24 × 10^−10^
NRN1_chr6_6004375_6004376	0.983253588516746	4.81 × 10^−9^
NRN1_chr6_6004428_6004429	0.982456140350877	2.35 × 10^−9^
NRN1_chr6_6004444_6004445	0.988038277511962	5.4 × 10^−11^
**NRN1** **_chr6_6004463_6004464**	**0.994417862838915**	**7.46 × 10^−12^**
NRN1_chr6_6004483_6004484	0.992025518767341	2.88 × 10^−11^
P14ARF_chr9_21968483_21968484	0.933811802232855	0.00000128
**P14** **ARF** **_chr9_21968504_21968505**	**0.937001594896332**	**0.000000174**
P14ARF_chr9_21968508_21968509	0.931419457735247	0.000000115
P14ARF_chr9_21968510_21968511	0.888357256778309	0.00000193
P14ARF_chr9_21968512_21968513	0.934609250398724	0.000000286
P14ARF_chr9_21968538_21968539	0.921850083230121	0.0000222
MEIS1_chr2_66667411_66667412	0.88755980861244	0.0000143
MEIS1_chr2_66667432_66667433	0.868421052631579	0.0000118
MEIS1_chr2_66667435_66667436	0.878787878787879	0.0000102
MEIS1_chr2_66667453_66667454	0.890749601275917	0.00000876
**MEIS1_chr2_66667460_66667461**	**0.891547049234238**	**0.00000827**

Bold marker used for MS-PCR detection; The classifier of the AUC analysis was methylation ratio of biomarkers in each sample.

**Table 3:** The diagnostic performance of different techniques in cohort 4.

Variables	Diagnostic Tool		Cytology		Fish	
	+	−	+	−	+	−
**UBC +**	38	6	16	28	30	14
**UBC −**	4	34	4	34	3	35
**Sensitivity**	0.864		0.364		0.682	
**Specificity**	0.895		0.895		0.921	
**PPV**	0.905		0.800		0.909	
**NPV**	0.850		0.548		0.714	

## Data Availability

The NGS data is available at https://www.ncbi.nlm.nih.gov/sra/PRJNA791014 (accessed on 18 February 2020). Accession to cite for these SRA data: PRJNA791014. The raw data will be released for public access upon acceptance of this publication.

## Supplementary Materials

The following supporting information can be downloaded at: https://www.mdpi.com/article/10.3390/cancers15030615/s1. Figure S1: The ROC curve of NRN1 plus P14ARF in cohort 2; Figure S2: The ROC curve of NRN1 plus P14ARF in cohort 2; Figure S3: The box plot and AUC of single NRN1 methylation biomarker in cohort 3 by detecting methylation regions; Figure S4: The box plot of 2 SNP biomarkers in cohort 3; Table S1. Clinical and pathological data of the cohort 1 and cohort 2; Table S2. Primers for different methylation regions; Table S3. The adjusted *p* value of different SNPs in Hunan multicenter cohort; Table S4. Primers for different SNP sites; Table S5. The NGS results and Δct values of two SNPs in cohort 3.

## Abbreviations

UBC: urothelial bladder carcinoma; NMIBC: non-muscle invasive bladder cancer; MIBC: muscle invasive bladder cancer; CT: computed tomography; XYH: Xiangya Hospital; SXYH: second Xiangya Hospital; HPPH: Hunan Provincial People’s Hospital; HCH: Hunan Cancer Hospital; BJH: Beijing Hospital; AUC: area under the curve; PPV: positive predictive value; NPV: negative predictive value; IQR: interquartile range.
