# Supplementary material for: A Novel Methylation Marker NRN1 plus TERT and FGFR3 Mutation Using Urine Sediment Enables the Detection of Urothelial Bladder Carcinoma
Source: Cancers (Basel). 2023 Jan 19;15(3):615. doi: 10.3390/cancers15030615 (PMC9913436; doi:10.3390/cancers15030615)
Supplement: Supplementary file 1 [file cancers-15-00615-s001.zip › Table S1.Clinical and pathological data of the cohort 1 and cohort 2.pdf]

Table S1. Clinical and pathological data of the cohort 1 and cohort 2

	Cohort 1		Cohort 2	
	UBC	Control	UBC	Control
Sample Size	33	19	38	31
Age, Years, Median (IQR)	63 (51-75)	61 (53-70)	59 (50-68)	57 (48-73)
Gender				
Male	23	14	27	25
Female	10	5	11	6
Smoking History				
Smoker	20	11	22	27
Non smokers	13	8	16	4
Stage				
Ta	8	/	10	/
T1	5		5	
T2	12	/	10	/
T3	5		10	
T4	3		3	
Grade				
Low grade	9	/	14	/
High grade	24	/	24	/
Control Group	/		/	
Urolithiasis	/	15	/	26
Infection	/	4	/	5
