# Supplementary material for: A Novel Methylation Marker NRN1 plus TERT and FGFR3 Mutation Using Urine Sediment Enables the Detection of Urothelial Bladder Carcinoma
Source: Cancers (Basel). 2023 Jan 19;15(3):615. doi: 10.3390/cancers15030615 (PMC9913436; doi:10.3390/cancers15030615)
Supplement: Supplementary file 1 [file cancers-15-00615-s001.zip › Table S2.Primers for different methylation regions.pdf]

Table S2 Primers for different methylation region

Methylation region		Primer
PSMD14_2_chr2_162284029_162284030	F	TAATTTATTTGTTATAAATATGC
	R	ARCTTAAATATCATTAAAATAAAAA
	P	TGAGAAGTTTAGAAAATGYAGATTTT
AKAP13_chr15_86233219_86233220	F	ATTGGATTTTGGGTTTAATGC
	R	AACCAAACTAAAATCAAATATTTA
	P	AGYTATTGATTGGYTTTGTGATTTTT
ZNF184_chr6_27463176_27463177	F	AGYATTAGGGGATATATTTAATGC
	R	CTTTAAATTTTAAAATACATATRCAC
	P	TGATGAGTTAATGGGTGYAGYATATTAATA
cg16966315_chr7_149112317_149112318	F	TTATATAGATTTTGAGTTTATTGC
	R	TAATAAAAACAAAATTTRCTATATT
	P	CCAARCTAATCTCTAACTCCTAACCTC
NRN1_chr6_6004463_6004464	F	TTTTAAAATTTAGTGGYGAAAGAAGC
	R	TTTACACAGCACACACTCACACATGC
	P	TTTAAAATGCATTGATATTTTTTAAGGGGAATT
P14ARF_chr9_21968504_21968505	F	ATTTGGYGGAGGGYAGAGAAAGC
	R	TCACTCCRCACCAAATRCAAAAAAT
	P	TGRAGGGTTGRAAGAAGAAAATGAGTGT
MEIS1_chr2_66667460_66667461	F	AGATTTGGTTAGATTTTTGC
	R	CCACTTTACATAAAGCACATC
	P	AGCTGCTGAATTTATATGCTTTGT
ACTB	F	AGTGAGAAAGGGTGTAGTTTTGGGAG
	R	CCACAAAAAAATAACCCAAATAAATAACCCACT
	P	CCTCTTCTAATAACCACCTCCCTCCTTCCTAAC
