# Supplementary material for: A Novel Methylation Marker NRN1 plus TERT and FGFR3 Mutation Using Urine Sediment Enables the Detection of Urothelial Bladder Carcinoma
Source: Cancers (Basel). 2023 Jan 19;15(3):615. doi: 10.3390/cancers15030615 (PMC9913436; doi:10.3390/cancers15030615)
Supplement: Supplementary file 1 [file cancers-15-00615-s001.zip › Table S3.The adjusted p value of different SNPs in Hunan multicenter cohort.pdf]

Table S3 The adjusted *p* value of different SNPs in Hunan multicenter cohort

SNP site	*p* value	Adjust *p* value
**TERT.chr5_1295228_1295228_G_A**	**2.0609E-12**	**3.5035E-11**
**FGFR3.chr4_1803568_1803568_C_G**	**5.139E-08**	**8.2224E-07**
PIK3CA.chr3_178936091_178936091_G_A	0.0038193	0.05728948
TERT.chr5_1295250_1295250_G_A	0.01035685	0.14499593
PIK3CA.chr3_178936082_178936082_G_A	0.02036447	0.26473809
FGFR3.chr4_1806099_1806099_A_G	0.04255489	0.5106587
PIK3CA.chr3_178952085_178952085_A_G	0.25848535	1
FGFR3.chr4_1803564_1803564_C_T	0.36408059	1
HRAS.chr11_533874_533874_T_C	0.44650398	1
TERT.chr5_1295228_1295228_G_T	0.70625115	1
HRAS.chr11_534289_534289_C_T	1	1
FGFR3.chr4_1807890_1807890_A_T	1	1
TP53.chr17_7577099_7577099_C_G	1	1
TP53.chr17_7577570_7577570_C_T	1	1
TP53.chr17_7578389_7578389_G_T	1	1
HRAS.chr11_534286_534286_C_G	1	1
PIK3CA.chr3_178921553_178921553_T_A	1	1

Bold SNP sites were selected for model optimization
