# Supplementary material for: A Novel Methylation Marker NRN1 plus TERT and FGFR3 Mutation Using Urine Sediment Enables the Detection of Urothelial Bladder Carcinoma
Source: Cancers (Basel). 2023 Jan 19;15(3):615. doi: 10.3390/cancers15030615 (PMC9913436; doi:10.3390/cancers15030615)
Supplement: Supplementary file 1 [file cancers-15-00615-s001.zip › Table S4.Primers for different SNP sites.pdf]

Table S4 Primers for different SNP sites

SNP site		Seq 5'->3' (5'FAM,3'MGB)
TERT C228T	F	GAGGGGGTTGGGTCGGGGATTCGGA
	R	CCGAACACCCGTCCTACCCCTTCA
	P	TCGGGACGGGGCGGGGTTCGCG
FGFR3 p.S249C	F	GTGGCGGTGACCAAGTTGGG
	R	AGAGAGCAGAGCGATAGGCG
	P	TGGCTGAGGAGTTGGTGGTGGCG
GAPDH	F	CATCTTCTTTTGCGTCGCCAG
	R	GGAGAGAACAGTGAGCGCCT
	P	CGGGAGGCTAGGGACGGCCTGAAGGC
