# Supplementary material for: A Novel Methylation Marker NRN1 plus TERT and FGFR3 Mutation Using Urine Sediment Enables the Detection of Urothelial Bladder Carcinoma
Source: Cancers (Basel). 2023 Jan 19;15(3):615. doi: 10.3390/cancers15030615 (PMC9913436; doi:10.3390/cancers15030615)
Supplement: Supplementary file 1 [file cancers-15-00615-s001.zip › Table S5.The NGS results and añct values of two SNPs in cohort 3.pdf]

Table S5. The NGS results and Δct values of two SNPs in cohort 3

Benign control groups	TERT C228T		FGFR3 p.S249C		UBC tumor groups	TERT C228T		FGFR3 p.S249C	
	NGS results	Δ ct values	NGS results	Δ ct values		NGS results	Δ ct values	NGS results	Δ ct values
DZX00018	Negative	21.83	Negative	20.81	DZX00063	Positive	9.92	Negative	21.02
DZX00028	Negative	22.57	Negative	19.98	DZX00124	Positive	12.60	Negative	21.45
DZX00029	Positive	9.75	Negative	17.08	DZX00002	Neagtive	22.57	Negative	22.37
DZX00032	Negative	22.12	Negative	22.17	DZX00003	Positive	12.60	Negative	21.57
DZX00035	Negative	25.49	Negative	23.81	DZX00004	Positive	8.26	Negative	20.18
DZX00036	Negative	22.44	Negative	21.68	DZX00005	Positive	14.72	Negative	21.66
DZX00037	Negative	21.73	Positive	14.60	DZX00006	Positive	11.02	Negative	21.31
DZX00040	Negative	21.81	Negative	23.52	DZX00104	Positive	11.04	Negative	21.38
DZX00062	Negative	18.48	Negative	21.88	DZX00020	Positive	4.76	Positive	4.80
DZX00075	Negative	22.02	Negative	18.11	DZX00015	Positive	10.26	Negative	21.02
DZX00083	Negative	21.24	Negative	21.38	DZX00014	Positive	5.83	Positive	5.20
DZX00084	Negative	22.51	Negative	22.97	DZX00076	Positive	14.99	Negative	21.67
DZX00087	Negative	22.21	Negative	19.80	DZX00011	Positive	9.78	Negative	20.80
DZX00089	Negative	22.67	Negative	22.65	DZX00012	Positive	8.68	Negative	20.19
DZX00091	Negative	21.50	Negative	22.07	DZX00024	Positive	4.08	Positive	4.27
DZX00093	Negative	22.48	Negative	19.16	DZX00074	Positive	9.30	Negative	20.58
DZX00096	Negative	21.79	Negative	20.36	DZX00009	Positive	4.19	Positive	4.42
DZX00097	Negative	25.16	Negative	22.08	DZX00008	Positive	6.73	Positive	5.86
DZX00098	Negative	16.69	Negative	22.27	DZX00071	Positive	6.08	Positive	5.42
DZX00102	Negative	18.64	Negative	22.67	DZX00121	Positive	8.09	Positive	14.91
DZX00117	Negative	22.12	Negative	20.86	DZX00250	Neagtive	22.00	Negative	22.63
DZX00119	Negative	22.37	Negative	22.17	DZX00068	Positive	3.65	Positive	17.08
DZX00120	Negative	19.38	Negative	21.55	DZX00230	Neagtive	21.05	Positive	3.81
DZX00125	Negative	21.05	Negative	23.62	DZX00105	Positive	9.21	Negative	20.36
DZX00126	Negative	21.31	Negative	22.30	DZX00110	Positive	8.68	Negative	20.19
DZX00128	Negative	20.58	Negative	22.80	DZX00131	Positive	14.49	Negative	21.59
DZX00130	Negative	21.78	Negative	21.24	DZX00111	Positive	11.46	Negative	21.38
DZX00135	Negative	22.62	Negative	20.80	DZX00132	Positive	11.02	Negative	21.38
DZX00136	Negative	22.00	Negative	21.11	DZX00113	Positive	8.06	Positive	14.60
DZX00137	Negative	19.20	Negative	22.56	DZX00114	Positive	12.09	Negative	21.39
DZX00148	Negative	21.95	Negative	21.66	DZX00080	Positive	11.80	Negative	21.39
DZX00164	Negative	21.21	Negative	23.89	DZX00118	Positive	4.29	Positive	4.73
DZX00170	Negative	23.74	Negative	21.95	DZX00214	Positive	4.76	Positive	4.83
DZX00171	Negative	21.53	Negative	22.15	DZX00249	Neagtive	20.76	Negative	21.99
DZX00185	Negative	22.60	Negative	22.54	DZX00082	Positive	10.64	Negative	21.25
DZX00187	Negative	21.69	Negative	22.90	DZX00115	Positive	10.03	Negative	21.02
DZX00195	Negative	21.15	Negative	21.68	DZX00168	Positive	10.57	Negative	21.11
DZX00198	Positive	9.21	Negative	21.62	DZX00260	Neagtive	22.43	Negative	22.30
DZX00200	Negative	22.26	Negative	17.77	DZX00086	Positive	9.25	Negative	20.58
DZX00201	Positive	10.57	Negative	21.98	DZX00262	Neagtive	21.02	Positive	3.56

Benign control groups	TERT C228T		FGFR3 p.S249C		UBC tumor groups	TERT C228T		FGFR3 p.S249C	
	NGS results	Δ ct values	NGS results	Δ ct values		NGS results	Δ ct values	NGS results	Δ ct values
DZX00205	Negative	21.46	Negative	17.84	DZX00090	Positive	11.02	Negative	21.27
DZX00217	Negative	20.76	Negative	22.96	DZX00095	Positive	6.62	Positive	5.64
DZX00222	Negative	21.99	Negative	19.77	DZX00172	Positive	12.09	Negative	21.42
DZX00223	Negative	22.24	Negative	21.36	DZX00177	Positive	8.77	Negative	20.19
DZX00224	Negative	21.67	Negative	21.13	DZX00178	Positive	7.75	Positive	11.16
DZX00225	Negative	23.12	Negative	20.18	DZX00182	Positive	7.75	Positive	10.11
DZX00226	Negative	21.76	Negative	22.35	DZX00184	Positive	10.63	Negative	21.24
DZX00228	Negative	21.02	Negative	21.98	DZX00134	Positive	11.46	Negative	21.38
DZX00231	Negative	21.11	Negative	22.37	DZX00239	Neagtive	21.78	Positive	19.58
DZX00232	Negative	21.91	Negative	21.99	DZX00142	Positive	9.86	Negative	20.81
DZX00233	Negative	22.43	Negative	21.48	DZX00144	Positive	12.60	Negative	21.44
DZX00234	Negative	19.81	Negative	21.57	DZX00186	Positive	10.57	Negative	21.21
DZX00235	Negative	22.40	Negative	22.40	DZX00189	Positive	7.28	Positive	6.76
DZX00236	Negative	20.19	Negative	22.40	DZX00145	Positive	10.64	Negative	21.24
DZX00237	Negative	21.49	Negative	21.59	DZX00190	Positive	14.72	Negative	21.66
DZX00238	Negative	22.67	Negative	22.09	DZX00191	Positive	9.20	Negative	20.19
DZX00240	Negative	21.38	Negative	21.86	DZX00147	Positive	17.87	Negative	21.69
DZX00241	Negative	21.84	Negative	21.27	DZX00149	Positive	14.49	Negative	21.57
DZX00272	Negative	23.65	Negative	21.39	DZX00100	Neagtive	18.65	Negative	21.78
DZX00277	Negative	22.09	Negative	20.60	DZX00169	Positive	9.20	Negative	20.36
DZX00292	Negative	21.74	Negative	21.80	DZX00216	Positive	10.57	Negative	21.15
DZX00297	Negative	21.82	Negative	21.72	DZX00220	Neagtive	21.81	Positive	19.99
DZX00300	Negative	21.65	Negative	19.30	DZX00246	Neagtive	19.20	Negative	21.84
DZX00303	Negative	18.04	Negative	22.50	DZX00153	Positive	8.06	Positive	14.60
DZX00311	Negative	22.16	Negative	22.21	DZX00227	Neagtive	18.64	Negative	21.80
DZX00315	Negative	22.06	Negative	22.60	DZX00157	Neagtive	18.61	Negative	21.72
DZX00322	Negative	22.40	Negative	20.71	DZX00158	Positive	10.26	Negative	21.02
DZX00324	Negative	22.28	Negative	21.50	DZX00160	Positive	14.49	Negative	21.63
DZX00326	Negative	19.99	Negative	20.42	DZX00116	Positive	7.30	Positive	8.12
DZX00346	Negative	22.52	Negative	21.98	DZX00192	Positive	7.74	Positive	10.08
DZX00347	Negative	20.20	Negative	22.28	DZX00194	Positive	7.76	Positive	12.85
DZX00355	Negative	21.35	Negative	22.50	DZX00206	Positive	17.87	Negative	21.68
DZX00356	Negative	21.75	Negative	22.57	DZX00207	Positive	10.57	Negative	21.15
DZX00360	Negative	22.21	Negative	21.39	DZX00210	Positive	7.28	Positive	6.68
DZX00361	Negative	21.85	Negative	21.57	DZX00211	Positive	11.02	Negative	21.38
DZX00378	Negative	22.14	Negative	21.79	DZX00229	Neagtive	21.69	Positive	18.11
DZX00406	Negative	19.26	Negative	22.27	DZX00247	Neagtive	19.99	Negative	21.91
DZX00407	Negative	22.44	Negative	22.02	DZX00255	Neagtive	21.31	Negative	22.24
DZX00415	Negative	21.73	Negative	22.42	DZX00256	Positive	7.28	Positive	6.87
DZX00473	Negative	25.49	Negative	21.57	DZX00252	Neagtive	23.65	Negative	22.50
DZX00488	Negative	25.16	Negative	21.96	DZX00257	Neagtive	21.21	Negative	22.08

Benign control groups	TERT C228T		FGFR3 p.S249C		UBC tumor groups	TERT C228T		FGFR3 p.S249C	
	NGS results	Δ ct values	NGS results	Δ ct values		NGS results	Δ ct values	NGS results	Δ ct values
DZX00498	Positive	9.92	Negative	17.68	DZX00258	Neagtive	20.58	Negative	21.96
DZX00587	Positive	14.72	Negative	21.63	DZX00261	Neagtive	22.62	Negative	22.43
DZX00613	Positive	11.02	Positive	3.81	DZX00263	Neagtive	21.91	Negative	22.62
					DZX00483	Positive	9.75	Negative	20.58
					DZX00389	Positive	18.48	Negative	21.70
					DZX00417	Neagtive	18.64	Negative	21.36
					DZX00338	Neagtive	18.96	Negative	21.84
					DZX00358	Neagtive	19.20	Negative	21.65
					DZX00461	Neagtive	19.56	Negative	21.86
					DZX00276	Neagtive	19.26	Negative	21.88
					DZX00451	Neagtive	19.38	Negative	21.91
					DZX00316	Neagtive	19.99	Negative	21.14
					DZX00321	Neagtive	16.83	Negative	21.48
					DZX00298	Neagtive	20.19	Negative	21.87
					DZX00329	Neagtive	20.45	Negative	21.43
					DZX00266	Neagtive	20.58	Negative	21.19
					DZX00486	Neagtive	20.73	Negative	21.82
					DZX00337	Neagtive	20.59	Negative	21.99
					DZX00305	Neagtive	21.76	Positive	1.89
					DZX00376	Neagtive	21.02	Positive	3.81
					DZX00364	Neagtive	21.05	Positive	4.04
					DZX00310	Neagtive	21.34	Negative	22.07
					DZX00363	Neagtive	21.11	Negative	22.08
					DZX00340	Neagtive	21.21	Negative	22.09
					DZX00289	Neagtive	21.78	Negative	22.03
					DZX00431	Neagtive	21.24	Negative	22.15
					DZX00445	Neagtive	21.31	Negative	22.27
					DZX00372	Neagtive	21.93	Negative	22.65
					DZX00330	Neagtive	21.38	Positive	17.08
					DZX00280	Neagtive	22.73	Positive	17.68
					DZX00470	Neagtive	21.46	Positive	17.75
					DZX00281	Neagtive	21.59	Positive	17.84
					DZX00409	Neagtive	21.50	Positive	18.04
					DZX00269	Neagtive	21.53	Positive	18.42
					DZX00440	Neagtive	21.65	Positive	18.36
					DZX00436	Neagtive	23.69	Positive	18.62
					DZX00374	Neagtive	21.86	Positive	18.64
					DZX00388	Neagtive	21.73	Positive	18.50
					DZX00270	Neagtive	21.12	Positive	19.20
					DZX00283	Neagtive	21.78	Positive	19.71
					DZX00457	Neagtive	21.81	Positive	19.77

Benign control groups	TERT C228T		FGFR3 p.S249C		UBC tumor groups	TERT C228T		FGFR3 p.S249C	
	NGS results	Δ ct values	NGS results	Δ ct values		NGS results	Δ ct values	NGS results	Δ ct values
					DZX00448	Neagtive	21.79	Positive	19.64
					DZX00275	Neagtive	21.83	Positive	20.18
					DZX00282	Neagtive	21.47	Negative	22.57
					DZX00480	Neagtive	21.98	Negative	22.72
					DZX00371	Neagtive	21.84	Negative	21.43
					DZX00304	Neagtive	20.37	Negative	22.62
					DZX00362	Neagtive	21.91	Negative	23.66
					DZX00312	Neagtive	21.99	Negative	22.46
					DZX00397	Neagtive	21.76	Negative	20.74
					DZX00265	Neagtive	22.02	Negative	23.52
					DZX00366	Neagtive	22.12	Negative	23.62
					DZX00375	Neagtive	22.18	Negative	23.81
					DZX00325	Neagtive	22.21	Negative	23.06
					DZX00405	Neagtive	22.26	Negative	23.89
					DZX00313	Neagtive	22.58	Negative	22.28
					DZX00335	Neagtive	22.37	Negative	22.34
					DZX00438	Neagtive	22.43	Negative	22.30
					DZX00264	Neagtive	22.44	Negative	22.35
					DZX00420	Neagtive	22.29	Negative	22.54
					DZX00336	Neagtive	22.57	Negative	22.40
					DZX00413	Neagtive	22.67	Negative	22.55
					DZX00317	Neagtive	22.60	Negative	22.42
					DZX00285	Neagtive	22.71	Negative	22.29
					DZX00395	Neagtive	22.62	Negative	22.43
					DZX00352	Neagtive	22.67	Negative	22.14
					DZX00459	Neagtive	23.20	Negative	22.34
					DZX00279	Neagtive	23.65	Negative	22.50
					DZX00379	Neagtive	24.67	Negative	22.56
					DZX00290	Neagtive	23.09	Negative	21.88
					DZX00373	Neagtive	25.16	Negative	21.76
					DZX00369	Neagtive	25.18	Negative	22.06
					DZX00447	Neagtive	25.49	Negative	19.60
					DZX00348	Neagtive	25.92	Negative	21.05
					DZX00410	Neagtive	25.90	Negative	23.98
