# Supplementary figures and images for: A Novel Methylation Marker NRN1 plus TERT and FGFR3 Mutation Using Urine Sediment Enables the Detection of Urothelial Bladder Carcinoma
Source: Cancers (Basel). 2023 Jan 19;15(3):615. doi: 10.3390/cancers15030615 (PMC9913436; doi:10.3390/cancers15030615)

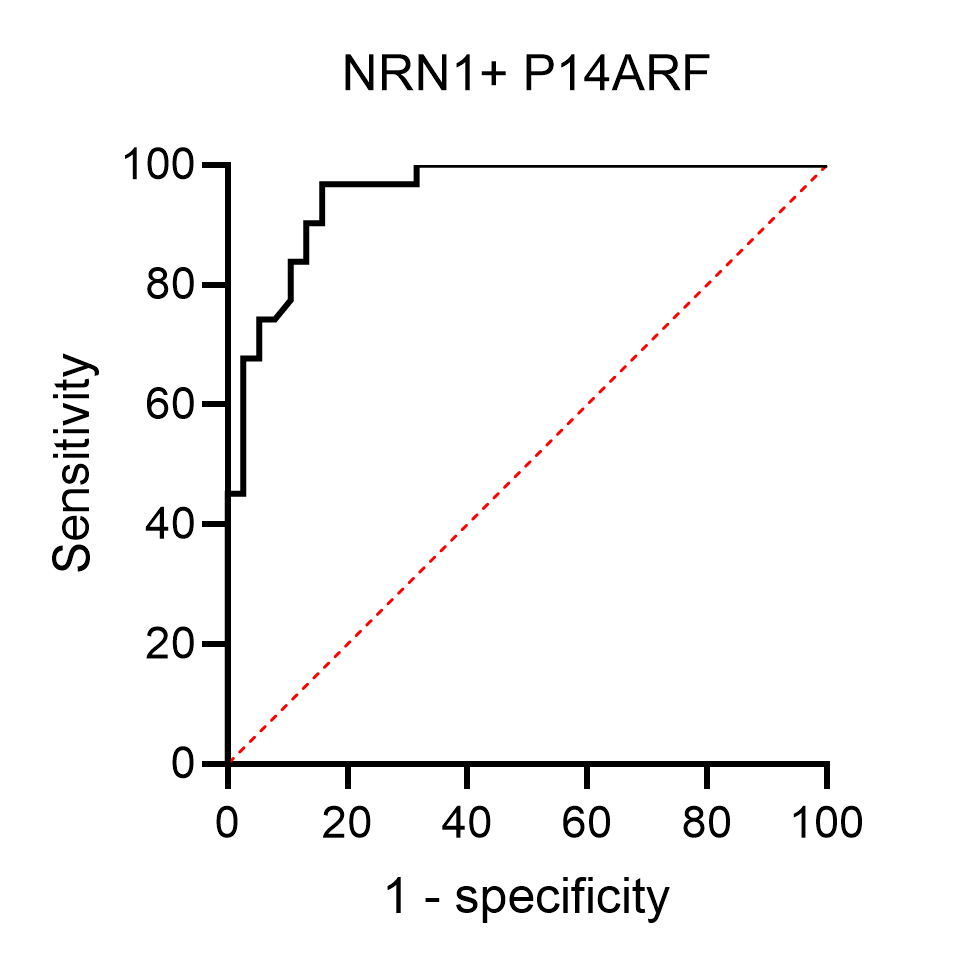

Supplement: Supplementary file 1 [file cancers-15-00615-s001.zip › Figure S1.The ROC curve of NRN1 plus P14ARF in cohort 2.tif]

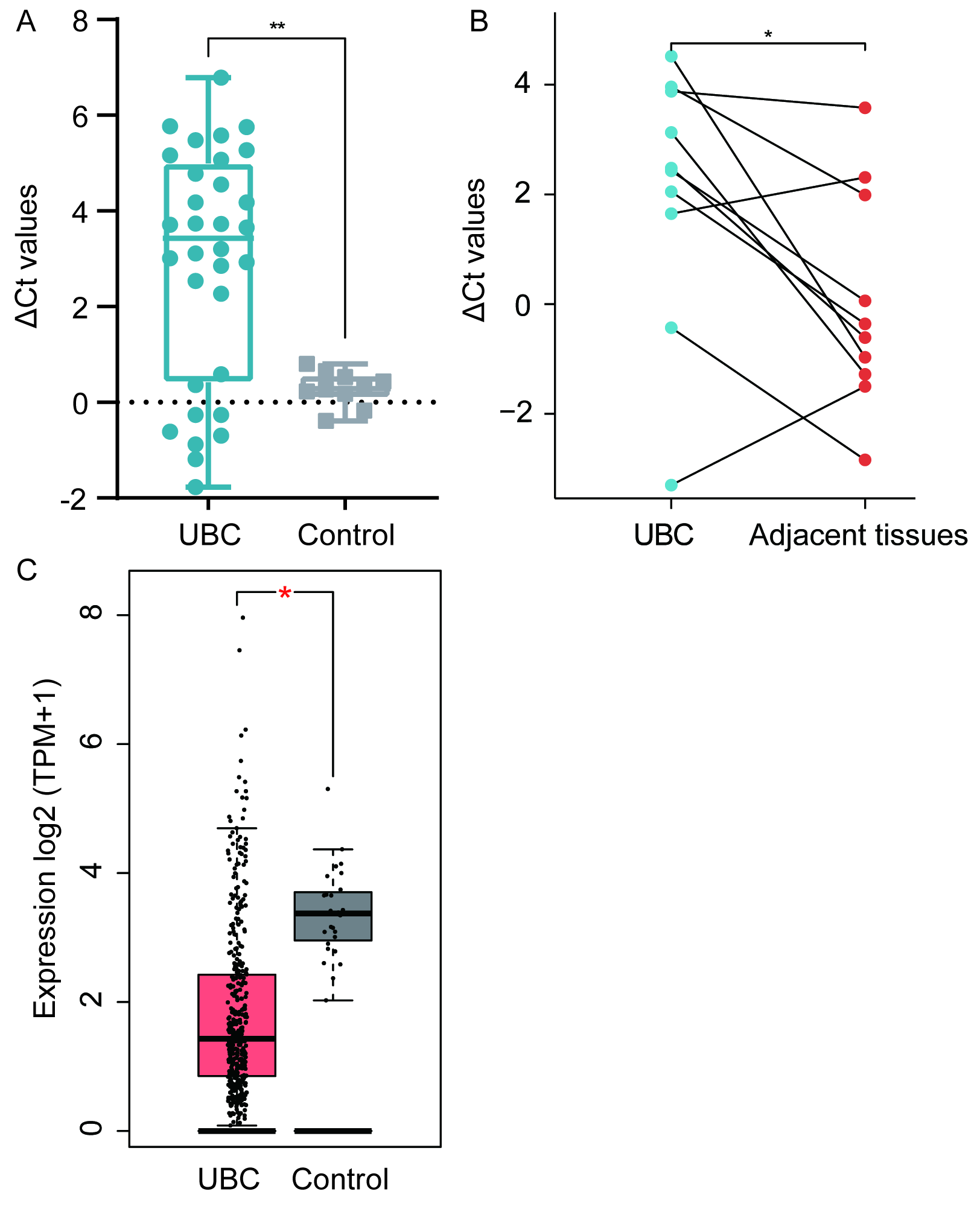

Supplement: Supplementary file 1 [file cancers-15-00615-s001.zip › Figure S2.The ROC curve of NRN1 plus P14ARF in cohort 2.tif]

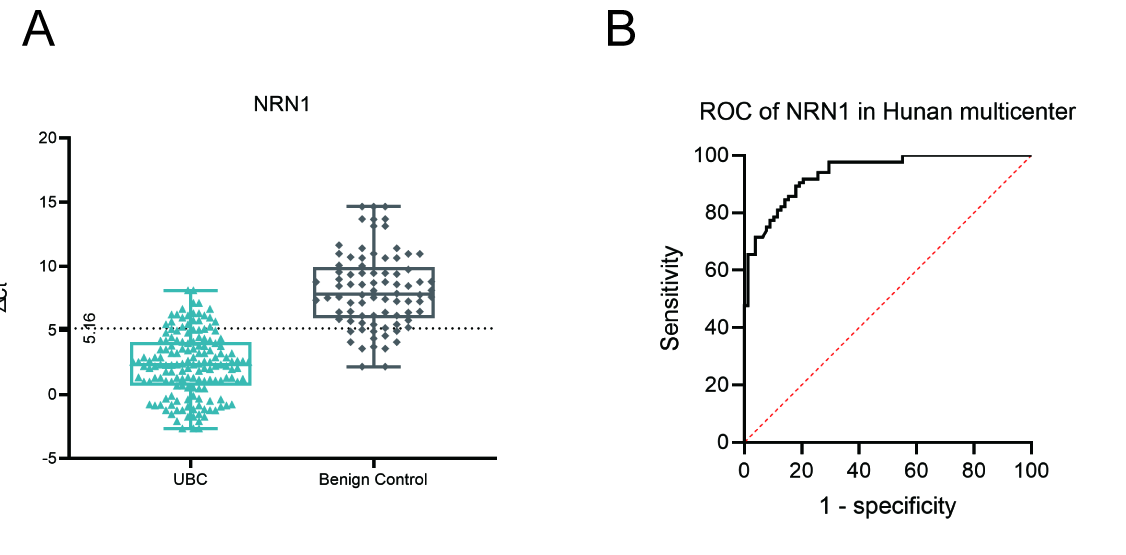

Supplement: Supplementary file 1 [file cancers-15-00615-s001.zip › Figure S3.The box plot and AUC of single NRN1 methylation biomarker in cohort 3 by detecting methylation regions.tif]

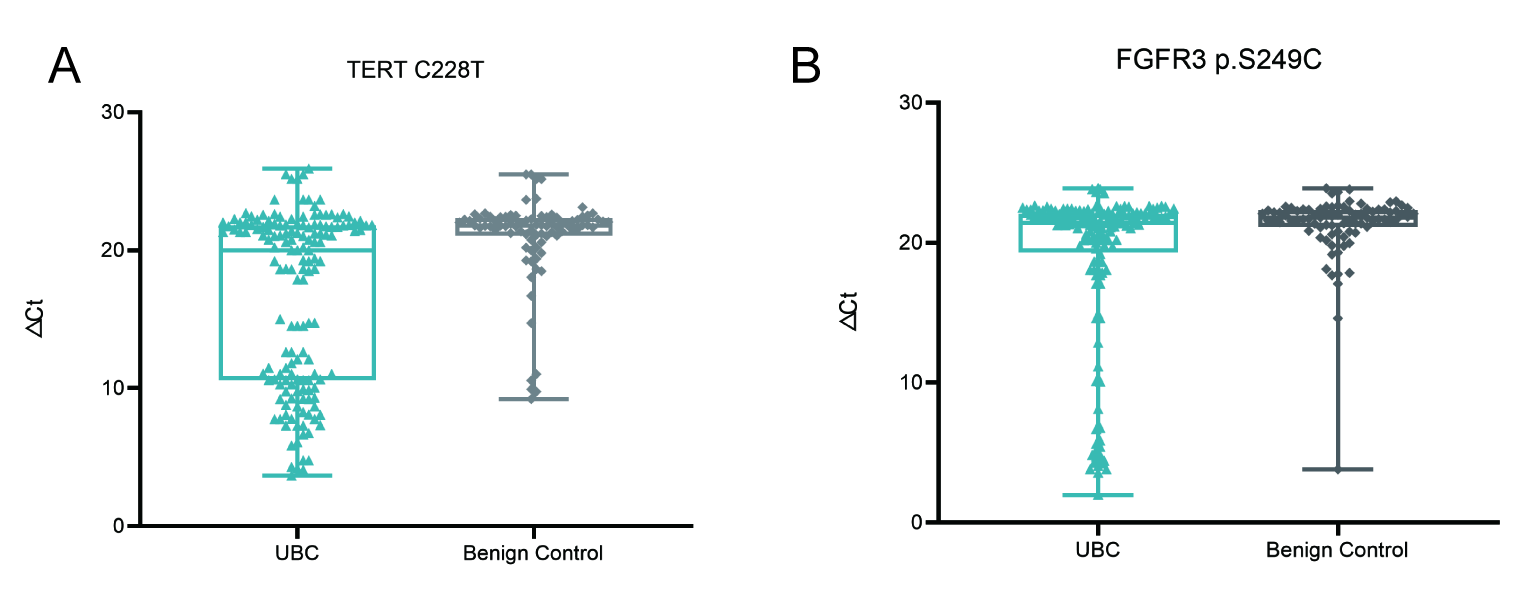

Supplement: Supplementary file 1 [file cancers-15-00615-s001.zip › Figure S4.The box plot of 2 SNP biomarkers in cohort 3.tif]
